# Transitions Pop-ups: Co-designing client-centred support for disabled youth transitioning to adult life

**DOI:** 10.3389/fresc.2024.1286875

**Published:** 2024-01-23

**Authors:** Yukari Seko, Anna Oh, Laura Thompson, Laura R. Bowman, C. J. Curran

**Affiliations:** ^1^ School of Professional Communication, Ryerson University, Toronto, ON, Canada; ^2^ Bloorview Research Institute, Toronto, ON, Canada; ^3^ Holland Bloorview Kids Rehabilitation Hospital, Toronto, ON, Canada; ^4^ London Health Sciences Centre, London, ON, Canada

**Keywords:** *Transitions Pop-ups*, transition to adulthood, pediatric rehabilitation, design thinking, human-centred design

## Abstract

**Background:**

When transitioning to adulthood, youth with disabilities and their families face many service gaps. Successful inter-agency collaborations can promote family-centred, inclusive transition support amenable to personal choice and health conditions. This paper reports the 3-year co-design process of an innovative transition service that links a pediatric hospital and adult service agencies and addresses key areas of transition preparedness with joint accountability.

**Methods:**

A team of pediatric rehabilitation professionals, adult service providers, young adults with disabilities and their families, and researchers engaged in a co-design process over three years. Following a design thinking (DT) framework, the team went through an iterative process of Empathize. Define, Ideation, Prototyping, and Testing phases. The trial-and-error process allowed for deeper reflection and an opportunity to pivot the design.

**Results:**

The co-design yielded *Transitions Pop-ups*, a nimble service model that can “pop up” at critical times and places to meet clients’ urgent and emergent transition-related needs. Two pilot sessions were conducted at the testing phase with adult service agencies. The final model included five key elements: (1) community partnership; (2) targeted information sharing; (3) peer mentoring; (4) action (on-the-spot completion of a key transition task/activity such as submitting an adult funding application); and (5) warm handover.

**Conclusion:**

The co-design process highlighted the importance of open communication and iterative prototype testing as a means for trialing new ideas and clarifying the intent of the project. The DT framework optimally facilitated the co-development of a contextually relevant and sustainable service model for pediatric rehabilitation clients and families.

## 1 Introduction

Transition from childhood to adulthood is a dynamic and multidimensional process. Once passing the age of majority, young adults often face societal expectations to go through educational, vocational, and legal status changes, take on new roles and responsibilities, and forge new relationships. For youth with disabilities, the transition process involves additional tasks and considerations associated with the move from pediatric to adult-oriented healthcare and social services. In Canada, young people transition from pediatric to adult healthcare systems by the age of 19. In contrast to pediatric services in which one or two main organizations provide holistic and developmentally appropriate care, adult services tend to be more dispersed and are managed by a wide range of specialists including primary care, hospital-based services, and community organizations with little formal coordination among them (1). Adult service users (individuals with disabilities and their families and other supporters) are expected to navigate the complex system on their own as autonomous individuals with full decision-making capacity, which might not always reflect the interdependent nature of disabled youth and their families’ lives (2).

The gaps in transition support for youth with disabilities and their families have been widely recognized. Some of the key obstacles identified in the literature include limited funding and services for adults with disabilities (3), the scarcity of coordinated, holistic, and life-course planning (4), and the lack of inter-sectoral care coordination that would facilitate a smooth transition (5). Suboptimal transition management may contribute to poor health outcomes including the risk of preventable complications and inappropriate reliance on emergency health services (6). Given the increasing number and diversities of youth with disabilities transitioning to adult services, researchers and service providers have also emphasized the importance of addressing a wide spectrum of life transitions (e.g., educational, vocational, and social participation opportunities) beyond clinical transfer (5).

In Canada, a national guideline for transition calls for the removal of barriers to inter-agency collaboration to promote family-centred, inclusive support amenable to personal choice and health conditions (2). A small but growing body of evidence suggests that a coordinated, “warm” handover to adult service providers can increase continuity of care and health service utilization (7, 8). Continuity of health service utilization reportedly reduced intensive re-engagement in the health system after reaching a point of crisis (8). The national guideline also emphasizes that design and delivery of transition services should involve multiple stakeholders including pediatric and adult care providers, policy makers, researchers, government agencies, and most importantly, youth with disabilities and their families.

Recently, the application of human-centred design (HCD) in healthcare settings has been growing exponentially with the call for client-centred care. HCD refers to a collaborative, people-centered, and interactive approach for designing products, services, or systems and is considered particularly effective when solving complex real-life challenges (9). A review by Göttgens and Oertelt-Prigione (10) identified 82 studies published between 2000 and 2020 that employed HCD approaches across various areas of health innovation. Among various HCD methods, design thinking (DT) emphasizes developing empathy for users and leverages end-user insights through an iterative process of empathize, define, ideate, prototype, and test. Through empathizing and defining, the designers first gain a deep understanding of what users really want and need to create an accurate problem statement. Ideation encourages the various possible ideas to choose from, not just looking for the best single solution. In prototyping and testing phases, the selected solutions are put to the users for them to test and provide feedback (11). DT was reportedly effective in having end users as design partners who engage in the entire design process, including feedback, idea generation, and decision-making (10).

To date, limited literature exists regarding the use of HCD in designing transition support services, with a particular scarcity of detailed description on the collaborative process in its entirety (12). Although design researchers have strived to create an inclusive co-design mechanism to meaningfully engage with end-users with diverse cognitive and physical abilities (13), the breadth and effectiveness of the HCD in designing transition services remains largely unknown. One notable exception is a study by Fortune et al. (14) that employed DT to co-design resources for young adults with cerebral palsy in transition to adult care. However, while this article provides rich descriptions of the initial phases of the co-design process, it ends at the prototyping stage without delineating whether and how prototypes were tested or implemented in actual service.

The goal of this paper is to describe the co-design process of *Transitions Pop-ups,* an innovative client-centred transition support service that links a pediatric hospital and local adult services to optimally support young adults and their families in transition to adulthood. We describe the process in which the *Transitions Pop-ups* model was co-designed by pediatric rehabilitation professionals, adult service providers, youth with disabilities and their families through an interactive and iterative design cycle over three years. In response to the call for transparency in reporting the co-design process (12), we report guiding principles underlying this inter-agency service model, facilitators and barriers encountered in the co-design process, and lessons learned. This knowledge can contribute to the growing body of HCD methods in healthcare practice and strengthen the evidence base for client-centred transition support in pediatric rehabilitation.

## 2 Project background

The project was conducted at Holland Bloorview Kids Rehabilitation Hospital (HBKRH) in Toronto, Canada, as part of the hospital's Transitions Strategy, a multi-year initiative to support a meaningful transition to adult life for clients graduating from the pediatric services. The Transitions Strategy aimed to enhance existing services and design new evidence-informed programs in partnership with young clients, families, and adult services (15). The objective of the co-design initiative was to create a scalable and sustainable service model to support collaboration between pediatric and adult services. To maximize involvement of hospital clients and families as “design partners” (10), the DT method was used to guide the entire process. One final prototype generated from the prototyping stage went through the testing phase to assess its feasibility in real-life situations. The service model was named *Transitions Pop-ups* and has been implemented as part of regular service at the HBKRH since 2019.

## 3 The design team

The overall project was led by a team of two occupational therapists with a combined 15 years experience in pediatric rehabilitation, system navigation, research, and solution-focused practice (LT&LB) and the Transitions Strategy Director (CC). Two researchers (YS and AO) conducted a program evaluation of the activities, discussions, and decisions. Other contributing team members included youth facilitators,^1^ family leaders,^2^ pediatric hospital clinicians (nurse practitioners, nurses, occupational therapists, therapeutic recreation specialists, social workers), and service providers from local adult services. These team members played essential roles throughout the co-design process, including co-facilitation of the empathize & define and ideation phases, acting in the prototyping phase (live scale modeling), and testing the service delivery model. Representatives from the Rotman School of Management (University of Toronto) NeXus Team supported the empathize & define phase. Consultants from Ontario's Ministry of Health and Long-term Care Business Innovation Office (MOHBIO) provided expertise in HCD and project management support at the ideation and prototyping phases. Overall, there were more than 100 individuals involved in the co-design process.

## 4 Design process

**Figure 1:**
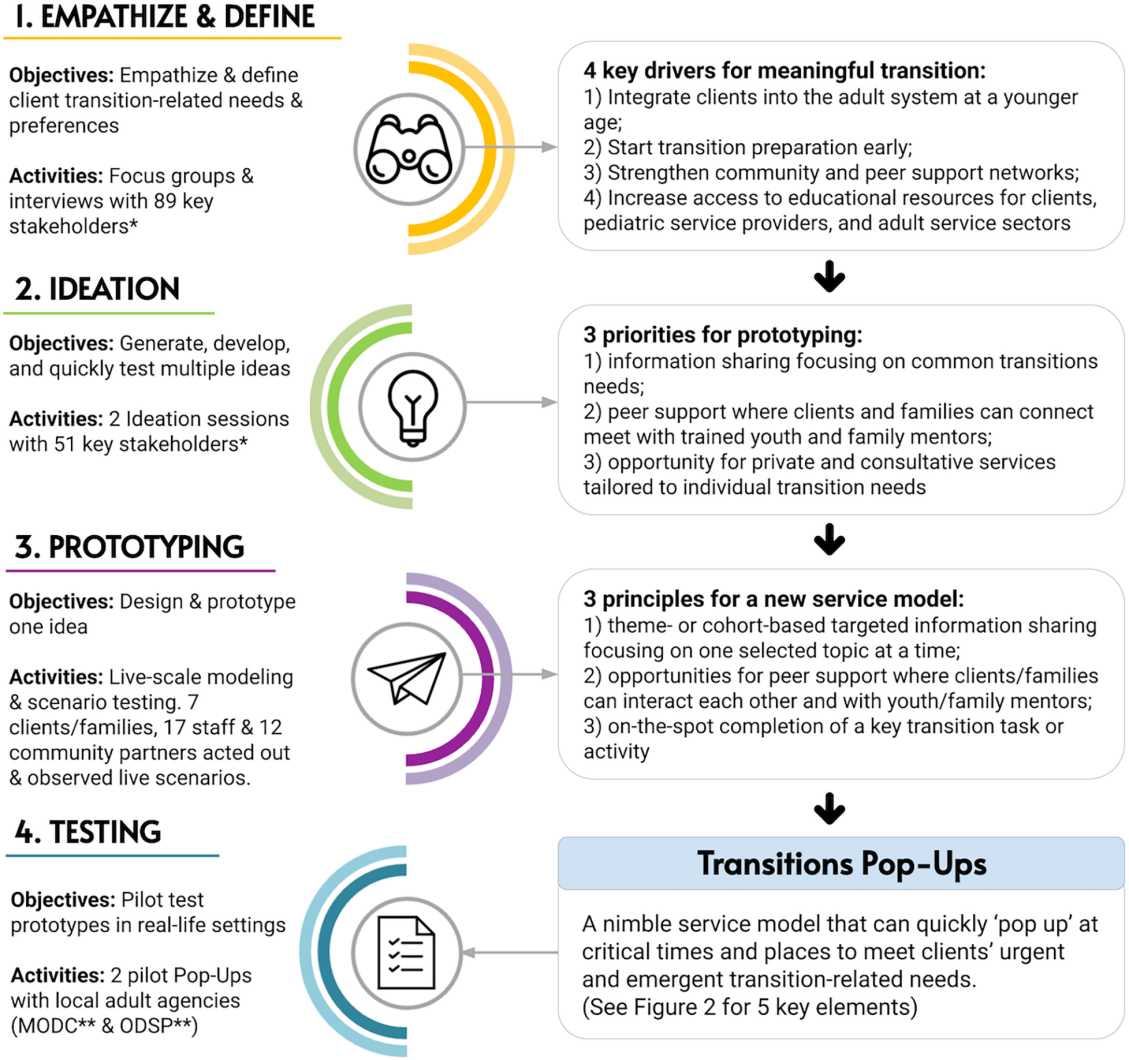
Human-centered design phases and activities in the *Transitions Pop-ups* design process. *Stakeholders including clients/families, hospital staff & community partners; **MODC, March of Dimes Canada; ODSP, Ontario Disability Support Program.

In what follows, we describe the four phases of the co-design process (Figure 1). Data for this article were retrieved from multiple sources including strategic planning documents, meeting minutes, post-session participant feedback forms, session fieldnotes, and staff debrief documents collected over three years.

### 4.1 Empathize & define phase

The first phase of DT involves building empathy with end users and gaining in-depth understanding of their needs and priorities (10). While the conventional DT framework separates “empathy” and “define” phases, we deliberately merged them into one. This approach allowed us to empathically engage with clients and families, collaborating to define problems and opportunities. In 2017, our team conducted a series of in-person interviews and focus group sessions to collaboratively explore the pre- and post-pediatric experiences of clients and families. For this stage, the third and fourth authors (both Occupational Therapists) recruited youth with disabilities and their families as part of the Transitions Strategy's outreach plan. Recruitment methods included advertisements through the hospital's Family Resource Centre and word-of-mouth referral. Identifying key service providers in the community involved an environmental scan and leveraging the authors’ professional networks.

Participants had the option to be interviewed either over the phone or in person. During in-person interviews, participants could choose to speak, write and/or have their answers transcribed in real time by the interviewer. In total, 89 participants took part, consisting of 30 former and current clients and/or their families from HBKRH, 27 HBKRH staff members, and 32 providers of local adult services. The young clients who participated in the project spanned ages 15–29, including both transition-aged clients (meeting the hospital's criteria of 15–21 years old) and those who had already undergone the transition process and wished to reflect on this period. Our participant selection purposefully represented the broad spectrum of HBKRH's disability and lived experience demographics, encompassing various physical, developmental, and/or neurological disabilities, as well as different educational, vocational, and social needs and aspirations for adult life.

Themes emerged from the interviews and focus groups, highlighting frustration with service gaps and a desire for a seamless, coordinated transition. Participants also noted the importance of developing self-management capacities among clients and families as a crucial factor for a smooth transition. The facilitators synthesized these themes into four key drivers for a meaningful transition: (1) helping clients to integrate into the adult system at a younger age; (2) starting transition preparation early; (3) strengthening community and peer support networks; and (4) increasing access to educational resources for clients, pediatric service providers, and adult service providers.

### 4.2 Ideation phase

Following the Empathize & Define phase, the team conducted two ideation sessions. In the DT framework, ideation is the process of generating, developing, and quickly testing as many ideas as possible (10). In the first ideation session, key stakeholders reviewed the aforementioned four key drivers for successful transition and discussed multiple ideas for a new service delivery model. In the second session participants further explored the ideas and created a series of small-scale prototypes. Two team members (LT and LB) led these sessions. In both ideation sessions, accommodated communication was offered to participants according to their developmental levels and communication preferences, including the use of Augmented and Alternative Communication (AAC) devices. Interpreters were available if needed for different languages, including American Sign Language.

#### 4.2.1 Ideation session 1

The first ideation session took place in January 2018 convening eight HBKRH clients, eight family members, 19 HBKRH staff (comprising service providers and leadership), and 16 collaborators from local adult services. Initially, participants reviewed the four key drivers that emerged from the Empathize & Define phase. Subsequently, they delved into the “pain points” experienced by clients and families, which encompassed feelings of being overwhelmed when introduced to adult services, a sense of disconnect from peers undergoing similar experiences, and a desire for opportunities to develop essential life skills before transitioning from pediatric services.

Participants were divided into six groups of 7–8 members and one facilitator. Participants were encouraged to think about how partnerships with the adult sector could help pediatric clients and families better prepare for transition, write down as many ideas as possible on sticky notes, and share their notes with other group members. Following the small group discussion, a larger group discussion ensued, allowing for the exchange of diverse stakeholder perspectives and feedback on individual ideas. Following the session, the facilitators collated and summarized the findings to present back to the group during the subsequent ideation session.

#### 4.2.2 Ideation session 2

The second ideation session occurred in April 2018 with 51 stakeholders consisting of 16 HBKRH clients and their family members, 19 HBKRH staff, and 16 collaborators from local adult services. While most participants from the first ideation session were able to attend the second session, a few opted out due to scheduling conflicts and other commitments. Consequently, there was a significant overlap of representatives from the initial session along with the inclusion of new participants.

During this session, participants were divided into six groups of 7–9 people, tasked with swiftly devising a small-scale prototype for a service/program aimed at addressing themes identified in the ideation session 1. Participants were encouraged to suspend judgment and expand on wild, out of the box ideas by drawing, in words, through songs and acting, arts and craft materials, and costumes. A member of the design team facilitated discussions within each small group.

Each group underwent two rounds of rapid prototyping and shared their ideas with the larger group. Emphasizing empathy towards service users and providers, participants were encouraged to consider their own feelings during the experience and ways to foster connections between the pediatric and adult systems. One lead facilitator guided a discussion among the larger group, while the other documented participants’ feedback, insights, and feelings about each prototype.

### 4.3 Prototyping phase: live scale modeling and scenario testing

Following the two ideation sessions, two team members (LT & LB) synthesized participant feedback and fieldnotes, revisiting four key transition drivers from the Empathize & Define phase. The team distilled this information into three guiding principles for prototyping a large-scale transition service: (1) information sharing focusing on common transition needs; (2) peer support where clients and families can connect with trained youth and family mentors; and (3) opportunity for consultative services tailored to individual transition needs. This led to the creation of a prototype: a mobile transitions service adaptable to various locations across the city.

In August 2018, seventeen hospital staff, twelve adult care professionals, and seven client and family members participated in live-scale prototyping. The set-up mirrored a “mobile fair,” consisting of multiple booths representing adult agencies specialized in areas such as independent living, attendant care, life skills, legal needs, and income support (target need: *information sharing*). A registration area with two transition facilitators was set up to help visitors navigate through the booths (target need: *consultative services*). There was also a private booth where visitors could access their health records on the spot and a lounge area for networking with youth/family mentors (target need: *peer support*).

Two simulated scenarios tested the live-scale prototype involving two youth-parent dyads (“simulators”). One dyad included a youth facilitator and hospital manager acting as a transition-aged youth and parent, and the other included a 15-year-old hospital client and her mother. The first scenario entailed a 17-year-old youth attending the mobile fair with her mother. The youth's goals included going to college, making friends, and living on her own after high school. In the second scenario, the 15-year-old youth and her mother were looking for information about community day programs available after high school and seeking support for navigating new routines and emotions that will arise during this major life change.

In each scenario, the simulators first met transition facilitators at the registration desk and engaged in a discussion about their hopes for adulthood. The facilitators then assisted the simulators with browsing related information, connecting with relevant adult services, and completing key transition tasks such as filling out adult funding applications. During the prototyping, a panel of stakeholders observed and provided on-the-spot feedback to the simulators, and the simulators improvisationally implemented the feedback. At the end of each scenario, the simulators were interviewed about their experiences and how the service could be more meaningful for clients and families. All participants (i.e., simulators, service providers at the booths, youth/family mentors who played transitions facilitators, and observers) were invited to complete a feedback survey about their experiences and share what was most effective about the prototype, potential challenges, and possible solutions.

Most participant feedback indicated that the live-scale prototyping was a one-of-a-kind opportunity to connect pediatric and adult service providers and co-design a service through authentic scenarios and role play. They valued several aspects (e.g., on-the-spot access to client health records to help simulators complete adult funding applications in real time). However, many participants felt the prototype was overwhelming for the families who received piles of pamphlets without knowing where to start and did not have time to connect with all adult providers of interest. Observers also suggested more innovation, seeking to avoid replicating existing information sessions offered at high schools (e.g., job fairs) or through HBKRH.

Following the live-scale prototyping, the team reviewed session feedback, noting the prototype's overwhelming nature despite the shared desire among participants for a “one-stop-shop”. Exploring where the mismatch of desire and prototype arose, the team revisited the lessons from the first two phases and revised the guiding principles. This process generated three refined principles that would characterize the new service delivery model: (1) targeted information sharing focusing on one selected topic at a time; (2) opportunities for meaningful peer support; and (3) on-the-spot completion of a key transition task/activity.

After several refinements, the team devised a new service model: *Transitions Pop-ups.* This nimble and agile service can “pop up” at critical times and locations to meet clients’ urgent and emergent transition needs. Just like pop-up retailing, the new model aimed to engage clients dynamically and generate a feeling of relevance and interactivity while maximizing resources. The new model targeted specific transition needs, highlighting the importance of authentic partnership with adult services for successful task completion.

### 4.4 Testing phase: two pilots with adult sector partners

Following the prototyping, the team conducted two pilot *Transitions Pop-ups* sessions to test the model in Spring and Fall 2019.

#### 4.4.1 Pilot 1: Try out an adult community program

The first pilot was conducted in partnership with the March of Dimes Canada's (MODC) Learning Independence for Future Empowerment (L.I.F.E) program. The L.I.F.E program supports young adults with disabilities (aged 15–30) in developing essential life and independence skills. The purpose of the event was for pediatric clients to try out a community program for adults and connect with adult service users. The program was a natural partner to test the *Transitions Pop-ups* model, as the MODC manager was a collaborator in the co-design process from the onset of the project.

The pilot took place over two sessions, with the first session taking place at HBKRH, and the second session being hosted at MODC. Eight clients and ten family members took part in the first session. The session focused on exploring participants’ preferred futures after high school and creating a vision of what a meaningful adult life might look like (e.g., activities, interests, roles). MODC staff shared a presentation on the L.I.F.E program and explained how the program can support clients in fulfilling their needs (principle 1: *targeted information sharing*). In the second session, three HBKRH clients and six family members joined the L.I.F.E program for a day. Clients participated in an independence-building activity with actual L.I.F.E. program participants (principle 3: *on-the-spot completion of a key transition task/activity*). Clients’ family members had opportunities to mingle with other family members and a family peer mentor in a separate room (principle 2: *meaningful peer support*). In the post-session feedback, participants reportedly found it helpful to meet with young adults and families who had experienced the transition to adult life, and to try out an adult community program before graduating high school.

#### 4.4.2 Pilot 2: connect with the primary adult funding agency

The second Transitions Pop-up was piloted in Fall 2019 with the Ontario Disability Support Program (ODSP). In Ontario, Canada, ODSP is the provincial funding program providing income and employment support for adults with disabilities based on medical and financial need. Although applying for ODSP marks a significant transition-related task for many clients and families, completing the lengthy funding application is often overwhelming during a time of complex life transition (1). Conversations with ODSP staff also revealed the desire for more opportunities to meaningfully interact with their clients upon funding application.

The pilot session took place at an ODSP office in Toronto with five HBKRH clients and eight family members. The goals of the event were for clients and families to increase their understanding of ODSP, complete an ODSP application form (if eligible), and learn the next steps in the ODSP application process and what to expect in the future. At the beginning of the session, a family leader shared her lived experience with applying for and receiving ODSP and what had been helpful (principle 2: *meaningful peer support*). Next, ODSP staff presented on the ODSP program, eligibility, and application timeline (principle 1: *targeted information sharing*). The session was purposefully designed to be interactive, with ODSP staff providing opportunities for individual consultation. At the end of the session, all eligible clients and families had completed an ODSP application with 1:1 support from ODSP caseworkers and HBKRH staff (principle 3: *on-the-spot completion of a key transition task/activity*). In the post-session feedback form, ODSP staff reported an increased awareness of clients’ and families’ needs and real-life challenges facing them with respect to ODSP applications.

**Figure 2:**
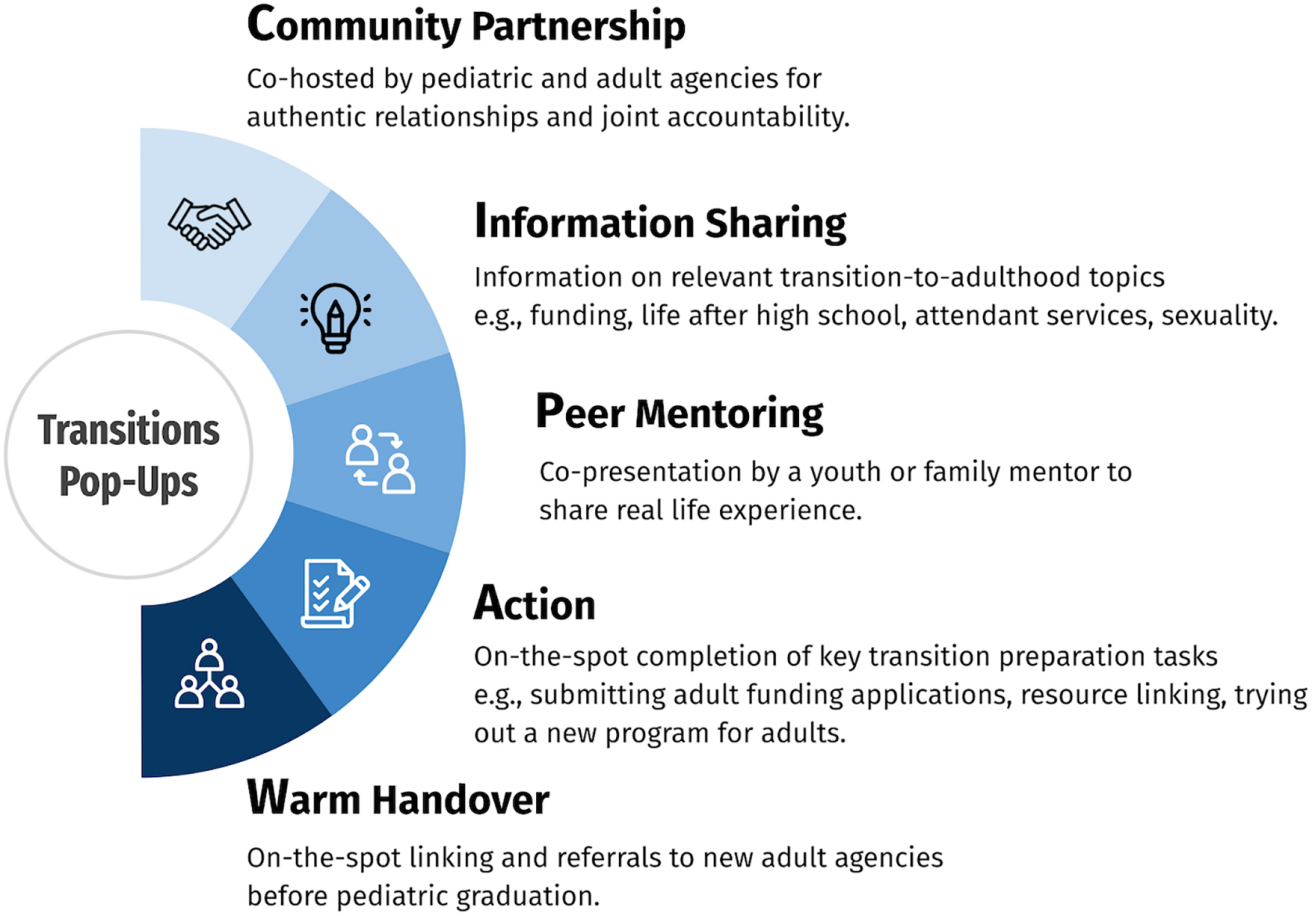
Five key elements of the final *Transitions Pop-ups* model.

The two pilot sessions at the testing phase were well received by participants. Following the two pilots, two additional elements were incorporated into the *Transitions Pop-ups* model to reflect client, family, and staff feedback. The five core elements of the model are shown in Figure 2.

## 5 Discussion

Over a three-year interactive co-design process, *Transitions Pop-ups* emerged as an innovative service model that can be tailored to diverse client needs, goals, and preferences. The model encourages proactive referrals, letting young clients explore adult programs before transitioning to adulthood. Since 2019, *Transitions Pop-ups* have expanded to 20 unique sessions that cover various topics including financial and legal support, health and wellness, life after high school, and personal care. All these topics originated from the co-design process and developed into individual sessions to cater optimally to participants’ needs. Many of these sessions have been conducted in partnership with local adult services, including both the MODC and ODSP. In 2021, the model was recognized as a leading practice by Accreditation Canada. As of 2023, HBKRH offers greater than 70 *Transitions Pop-ups* per year, on 20 discrete transition topics, with 1092 client/family attendances since 2019.

The Design Thinking (DT) methodology guided the entire process by actively involving multiple stakeholders, generating new ideas, and developing a service delivery model that can be implemented in regular service at HBKRH. Although we described the process sequentially, it felt fluid and messy, involving constant iterations and refinement of ideas. DT's fundamental tolerance for trial and error was invaluable to the co-design process. As previously mentioned, the initial live-scale prototype felt overwhelming for participants, despite it being developed through multiple iterations and dialogues. However, this “failure” highlighted the need to explore one transition-related task/activity at a time, rather than putting together vast information in a single physical space. The lesson learned emphasized the importance of embracing flexibility and remaining open to new ideas, rather than strictly adhering to one idea.

A key driver of success was positive and accountable partnerships forged between the pediatric hospital and local adult services. The design team established collegial relationships with local adult services, sharing a passion to facilitate seamless transitions. Early involvement of senior leadership significantly boosted the project's momentum, while donor funding supported the entire process. Most importantly, the constant involvement of stakeholders who had lived experience of disability, such as youth facilitators, family leaders, former and current clients and families, was pivotal in steering the co-design process towards success. It was vital to implement inclusive and adaptive communication methods to ensure diverse voices were expressed and heard, thereby expanding the project's reach and relevance within the community.

In terms of barriers, the co-design proved to be time- and labor-intensive. To optimize time, the core members synthesized and shared ideas between the sessions, yet maintaining transparency in the decision making process was not easy. Existing literature lacks guidance on maintaining transparency in large HCD processes like ours. To address this gap, our project incorporated ongoing program evaluation and meticulously documented each activity, discussion, and decision to maintain an extensive audit trail. This allowed the team to provide ongoing feedback to improve the co-design process and the service model. Lastly, addressing the heterogeneous experiences of transitions to adulthood posed challenges. While we focused on common transition-related tasks and experiences applicable to many of our clients/families (e.g., life skills programs for adults, ODSP funding applications, legal considerations, sexuality), it may not encompass unique individual experiences of youth with disabilities and their families. Rehabilitation practitioners who wish to integrate DT in their service design should aim for adaptability, recognizing the diversity within the experiences of those they serve. We suggest embracing a dual approach—attending to general needs while remaining receptive to the nuances of specific circumstances. This flexibility allows for a more inclusive and responsive service design, ensuring that the broader aspects cater to many while leaving room to address distinct needs.

## 6 Conclusion

Our co-design initiative has both design and intervention implications. From a design perspective, the DT provided a useful framework to engage service users and providers, and the process of iteration and open feedback was vital to optimize service design and delivery. We learned that assumptions of shared understanding can sometimes be misleading; the live-scale modeling prototype met all requested needs of the group, and yet it was not embraced by collaborators. This trial-and-error process allowed for a deeper reflection and an opportunity to pivot the design. From an intervention perspective, the *Transitions Pop-ups* model has been built through consultation with best available evidence, community collaboration, and lived experience, and can be implemented across transition-related services and programs. Future initiatives in pediatric rehabilitation can use DT as a means for trialing new ideas and clarifying the core intent of projects.

## Data Availability

The raw data supporting the conclusions of this article will be made available by the authors, without undue reservation.

## Footnotes

^1^Youth Facilitators (YFs) are peer service providers with childhood-onset disabilities with capacity to meet with clients individually, access patient health records, conduct clinical documentation, and make clinical referrals as needed. Since 2006, the role has been integrated in clinical teams at the HBKRH. For a detailed explanation of the youth facilitator role see Seko et al. (16).

^2^Family leaders are family members or caregivers with lived experience of supporting children/youth with disabilities who volunteer at the hospital.
